# Design and Synthesis of Novel *N*-Arylsulfonyl-3-(2-yl-ethanone)-6-methylindole Derivatives as Inhibitors of HIV-1 Replication

**DOI:** 10.3390/ph8020221

**Published:** 2015-05-08

**Authors:** Zhiping Che, Shengming Liu, Yuee Tian, Zhenjie Hu, Yingwu Chen, Genqiang Chen

**Affiliations:** Laboratory of Pharmaceutical Design & Synthesis, Department of Plant Protection, College of Forestry, Henan University of Science and Technology, Luoyang 471003, Henan, China

**Keywords:** *N*-arylsulfonyl-3-(2-yl-ethanone)-6-methylindoles, human immunodeficiency virus type-1, inhibitor

## Abstract

Seven novel *N*-arylsulfonyl-3-(2-yl-ethanone)-6-methylindole derivatives **4a**–**f** and **6** were readily synthesized and have been identified as inhibitors of human immunodeficiency virus type-1 (HIV-1) replication. Initial biological studies indicated that among these derivatives, *N*-(*p*-ethyl)phenylsulfonyl-3-[2-morpholinoethanone]-6-methylindole (**4f**) and *N*-(*p*-ethyl)phenylsulfonyl-3-[2-(5-phenyl-1,3,4-oxadiazole-2-yl-thio)ethanone]-6-methylindole (**6**) showed the most promising activity against HIV-1 replication. The effective concentration (EC_50_) and therapeutic index (TI) values of **4f** and **6** were 9.42/4.62 μM, and >49.77/66.95, respectively. The cytotoxicity of these compounds has also been assessed. No significant cytotoxicities were found for any of these compounds.

## 1. Introduction

**Figure 1:**
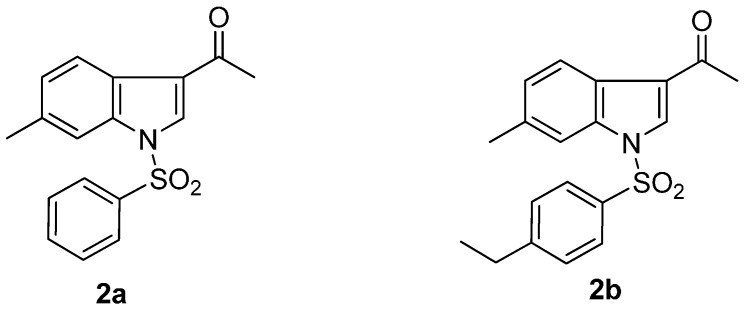
Structures of *N*-phenylsulfonyl-3-acetyl-6-methylindole (**2a**) and *N*-(*p*-ethyl)-phenylsulfonyl-3-acetyl-6-methylindole (**2b**).

**Scheme 1:**
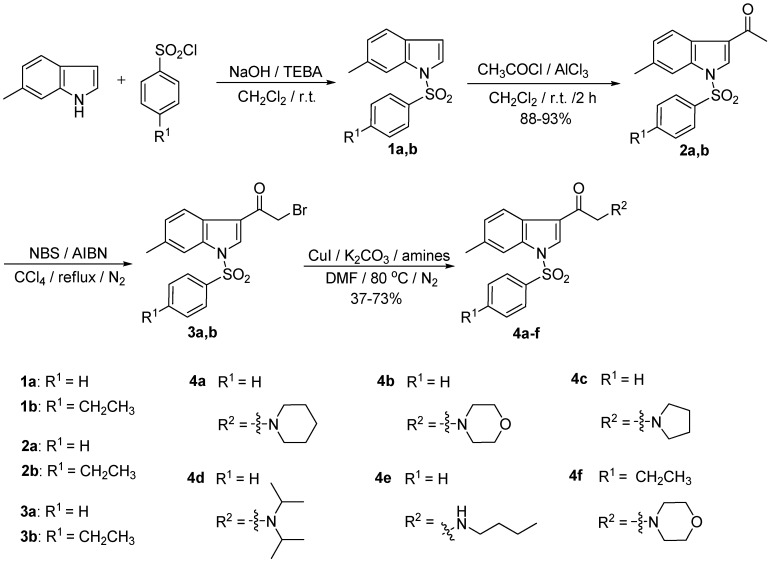
The synthetic route of compounds **4a**–**f**.

**Scheme 2:**
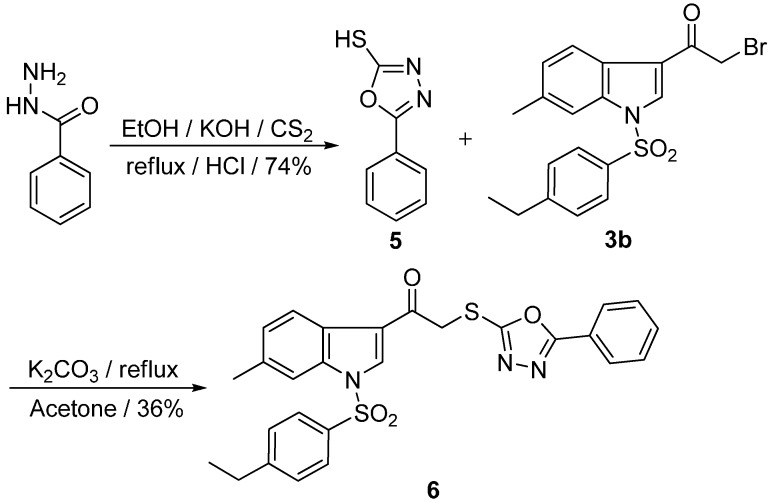
The synthetic route of compound **6**.

The use of chemotherapy to suppress replication of the human immunodeficiency virus (HIV) has transformed the face of acquired immunodeficiency syndrome (AIDS) in the developed world. Pronounced reductions in illness and death have been achieved and healthcare utilization has diminished. HIV therapy has also provided many new insights into the pathogenesis and the viral and cellular dynamics of HIV infection, but challenges remain. Treatment does not suppress HIV replication in all patients, and the emergence of drug-resistant virus hinders subsequent treatment. Chronic therapy can also result in toxicity. These challenges prompt the continued search for new drugs and new therapeutic strategies to control chronic viral replication [1,2,3,4]. Consequently, the design and synthesis of brand new, specific, efficacious, safe chemotherapeutic drugs for the prevention of the spread of HIV-1 infection is imperative. In our previous studies, *N*-arylsulfonylindoles and *N*-arylsulfonyl-3-acetylindoles have been demonstrated the significant anti-HIV-1 activity *in vitro* [5,6]. Particularly, *N*-phenylsulfonyl-3-acetyl-6-methylindole (**2a**, Figure 1) and *N*-(*p*-ethyl)phenylsulfonyl-3-acetyl-6-methylindole (**2b**, Figure 1) have shown the most potent anti-HIV-1 activity. This confirmed that introduction of the acetyl group at the 3-position of *N*-arylsulfonyl-6-methylindoles could generally lead to more potent analogs. As a continuation of the research program of our laboratory searching for anti-HIV-1 agents [7,8,9], we now report the design and synthesis of some novel *N*-arylsulfonyl-3-(2-yl-ethanone)-6-methylindoles derivatives **4a**–**f** and **6** (Scheme 1 and Scheme 2), which can specifically inhibit HIV-1 replication.

## 2. Results and Discussion

### 2.1. Chemistry

*N*-Arylsulfonyl-3-(2-yl-ethanone)-6-methylindole derivatives **4a**–**f** were synthesized as shown in Scheme 1. First, **1a**,**b** and **2a**,**b** were obtained as reported in an earlier paper [6]. Subsequently, the key intermediates, *N*-arylsulfonyl-3-(2-bromoethanone)-6-methylindoles **3a**,**b** were obtained by reaction of *N*-bromosuccinimide with 2,2'-azobisisobutyronitrile (AIBN) in a CCl_4_ solution under nitrogen. Compounds **3a,b** were used directly in the next step without further purification. Finally, **3a**,**b** reacted with the corresponding amines in the presence of cuprous iodide (CuI) and anhydrous potassium carbonate (K_2_CO_3_) at 80 °C to give **4a**–**f** in 37%–73% yields. The compounds were well characterized by ^1^H-NMR, ^13^C-NMR, m.p., and MS.

*N*-(*p*-Ethyl)phenylsulfonyl-3-[2-(5-phenyl-1,3,4-oxadiazole-2-yl-thio)ethanone]-6-methylindole (**6**) was synthesized as depicted in Scheme 2. Firstly, intramolecular cyclization of benzoylhydrazine with carbon disulfide and potassium hydroxide in the presence of ethanol resulted in 5-phenyl-1,3,4-oxadiazole-2-thiol (**5**) [10]. Subsequently, *N*-(*p*-ethyl)phenylsulfonyl-3-(2-bromoethanone)-6-methylindole (**3b**) was reacted with **5** in the presence of K_2_CO_3_ to afford **6** in 36% yield.

### 2.2. Biological Activity

At the outset, the title compounds of series **4a**–**f** and compound **6** were tested *in vitro* for their ability to inhibit HIV-1-induced cytopathogenicity, for cytotoxicity and anti-HIV-1 activity in acutely infected C8166 cells in comparison with 3'-azido-3'-deoxythymidine (AZT) used as a positive control (Table 1). All compounds tested could inhibit HIV-1 replication. However, none was as effective as AZT. Among them, compounds **4a**, **4d**, **4f** and **6** exhibited the most potent anti-HIV-1 activity, with EC_50_ values of 32.78, 17.94, 9.42 and 4.62 μM, and therapeutic index (TI) values of >15.38, 12.26, >49.77 and 66.95, respectively. Maximum activity were obtained with compounds **4f** and **6**, which were endowed with the highest potency (TI > 49.77 and = 66.95, respectively). However, compounds **4b**, **4c**, and **4e** only showed moderate activity (TI > 8.87, = 2.12 and = 1.46, respectively). The cytotoxicity of these compounds had also been assessed. No significant cytotoxicities were found for any of them.

Meanwhile, some preliminary structure-activity relationships (SAR) of **4a**–**f** and **6** were also observed. We examined the effect of substituents at the C-2 position of the acetyl by use of various amines and 5-phenyl-1,3,4-oxadiazole-2-thiol groups. It can be seen that the piperidinyl-substituted compound **4a** was generally more potent than the diisopropylamino-substituted compound **4d**, the morpholino-substituted compound **4b**, the pyrrolidino-substituted compound **4c** and the butylamino-substituted compound **4e**, if the R^1^ substituents at the C-4 position of the arylsulfonyl ring were the same. The cytotoxicity (CC_50_), anti-HIV-1 activity (EC_50_) and TI values of **4a**, **4d**, **4b**, **4c**, **4e** were >504.41/220.02/501.91/225.82/43.79 μM, 32.78/17.94/56.56/106.02/29.96 μM, and 15.38/12.26/8.87/2.12/1.46, respectively. Especially the TI value of **4a** was more than 10 times that of **4e**. Interestingly, variations at the C-4 position of the arylsulfonyl ring play an important role in HIV-1 replication inhibition. For example, the EC_50_ and TI values of **4b** and **4f** were 56.56/9.42 μM, and >8.87/>49.77, respectively.

It is worth noting that the insertion of an ethyl group at the *para-*position of the arylsulfonyl ring led to compound **4f** characterized by both low cytotoxicity (CC_50_ > 468.91 μM) and antiviral activity (TI > 49.77). A change in activity was observed for compound **4b** (EC_50_ = 56.56 μM, TI > 8.87) when compared with **4f** (EC_50_ = 9.42 μM, TI > 49.77). The results suggest that the substituent at R^1^ is an important feature for activity. Moreover, when the 5-phenyl-1,3,4-oxadiazole-2-thiol group was introduced at the C-2 position of **3b**, the corresponding compound **6** displayed the most potent anti-HIV-1 activity. The EC_50_ and TI values of **6** were 4.62 μM and 66.95.

**Table 1:** Anti-HIV-1 activity of *N*-arylsulfonyl-3-(2-yl-ethanone)-indoles derivatives **4a**–**f** and **6** *in vitro* ^a^.

Compounds	CC_50_^b^(µM)	EC_50_^c^(µM)	TI ^d^
**4a**	>504.41	32.78	>15.38
**4b**	>501.91	56.56	>8.87
**4c**	225.82	106.02	2.12
**4d**	220.02	17.94	12.26
**4e**	43.79	29.96	1.46
**4f**	>468.91	9.42	>49.77
**6**	309.31	4.62	66.95
**AZT ^e^**	4263.84	0.01212	351801.98

^a^ Values are means of two separate experiments. ^b^ CC_50_ (50% cytotoxic concentration), concentration of drug that causes 50% reduction in total C8166 cell number. ^c^ EC_50_ (50% effective concentration), concentration of drug that reduces syncytia formation by 50%. ^d^ In vitro therapeutic index (CC_50_ value/EC_50_ value). ^e^AZT was used as a positive control.

## 3. Experimental Section

### 3.1. General Information

All reagents and solvents were of reagent grade or purified according to standard methods before use. Analytical thin-layer chromatography (TLC) and preparative thin-layer chromatography (PTLC) were performed with silica gel plates using silica gel 60 GF_254_ (Qingdao Haiyang Chemical Co., Ltd., Qingdao, China). Melting points were determined on a digital melting-point apparatus and were uncorrected. ^1^H-NMR and ^13^C-NMR spectra were recorded at 300 MHz or 400 MHz or 500 MHz and 125 MHz respectively on a Bruker Avance DMX 300 MHz or 400 MHz or 500 MHz spectrophotometer in CDCl_3_ with tetra methyl silane (TMS) as an internal standard. Electrospray iontrap mass spectrometry (ESI-TRAP-MS) and mass spectra (EI-MS) were carried out with Bruker ESI-TRAP Esquire 3000 plus mass spectrometry instrument and a HP 5988 instrument, respectively. High-resolution mass spectra (HR-MS) were recorded with an IonSpec 4.7 T FTMS instrument.

### 3.2. General Procedure for the Synthesis of **4a**–**f**

Compounds **1a,b** and **2a,b** were prepared as reported in the earlier paper [6]. The structures of the compounds were well characterized by ^1^H-NMR, m.p., and MS.

*N-Phenylsulfonyl-6-methylindole* (**1a**): White solid, m.p.70–72 °C. ^1^H-NMR (300 MHz, CDCl_3_) *δ*: 7.81–7.88 (m, 3H), 7.41–7.49 (m, 5H), 7.04 (d, *J* = 6.9 Hz, 1H), 6.61 (s, 1H), 2.47 (s, 3H). EI-MS *m/z*: 271 (M^+^, 48).

*N-(p-Ethyl)phenylsulfonyl-6-methylindole* (**1b**): White solid, m.p. 84–86 °C. ^1^H-NMR (300 MHz, CDCl_3_) *δ*: 7.76–7.81 (m, 3H), 7.49 (s, 1H), 7.38 (d, *J* = 7.8 Hz, 1H), 7.03–7.25 (m, 3H), 6.59 (s, 1H), 2.61 (q, *J* = 7.2 Hz, 2H), 2.47 (s, 3H), 1.17 (t, *J* = 7.2 Hz, 3H). EI-MS *m/z*: 299 (M^+^, 42).

*N-Phenylsulfonyl-3-acetyl-6-methylindole* (**2a**): White solid, m.p. 220–221 °C. ^1^H-NMR (300 MHz, CDCl_3_) *δ*: 8.16 (d, *J* = 8.4 Hz, 1H), 8.14 (s, 1H), 7.93 (d, *J* = 7.5 Hz, 2H), 7.73 (s, 1H), 7.58–7.63 (m, 1H), 7.48–7.53 (m, 2H), 7.15 (d, *J* = 8.1 Hz, 1H), 2.56 (s, 3H, COCH_3_), 2.47 (s, 3H, CH_3_). ESI-TRAP-MS *m/z*: 314 ([M+H]^+^, 100).

*N-(p-Ethyl)phenylsulfonyl-3-acetyl-6-methylindole* (**2b**): White solid, m.p. 118–120 °C. ^1^H-NMR (400 MHz, CDCl_3_) *δ*: 8.17 (d, *J* = 8.4 Hz, 1H), 8.14 (s, 1H), 7.84 (d, *J* = 8.0 Hz, 2H), 7.74 (s, 1H), 7.30 (d, *J* = 8.0 Hz, 2H), 7.15 (d, *J* = 8.0 Hz, 1H), 2.66 (q, *J* = 7.6 Hz, 2H, CH_2_CH_3_), 2.55 (s, 3H, COCH_3_), 2.48 (s, 3H, CH_3_), 1.18 (t, *J* = 8.0 Hz, 3H, CH_2_CH_3_). EI-MS *m/z*: 341 (M^+^, 29).

A solution of *N*-arylsulfonyl-3-acetyl-6-methylindoles **2a,b** (1 mmol) in CCl_4_ (5 mL) was heated to reflux after which *N*-bromosuccinimide (1.1 mmol) and AIBN (8 mg) were admixed carefully and added in three portions under nitrogen. The mixture was refluxed for 20 min. Another portion of AIBN (4 mg) was then added. The mixture was kept at reflux for 3 h and then allowed to cool. The precipitated succinimide was filtered off and washed with hexane (4 × 10 mL). The combined filtrates were concentrated under reduced pressure to afford **3a,b**. The crude residues of **3a,b** were flash evaporated under reduced pressure with dry THF three times and used directly in the next step without further purification. These compounds are unstable on silica gel. Subsequently, to a mixture of **3a,b** (1 mmol), the corresponding amines (1 mmol), CuI (0.2 mmol) and anhydrous K_2_CO_3_ (2 mmol) in DMF (5 mL) were added with stirring at 80 °C under nitrogen. When the reaction was complete according to TLC analysis, the reaction mixture was cooled to room temperature, poured into ice water (20 mL), and extracted with EtOAc (30 mL × 3). Finally, the combined organic phase was washed by brine, dried over anhydrous Na_2_SO_4_, concentrated *in vacuo* and purifed by silica gel column chromatography to give the pure compounds **4a**–**f**. The structures of the compounds were well characterized by ^1^H-NMR, ^13^C-NMR, m.p., and MS.

*N-Phenylsulfonyl-3-[2-piperidinylethanone]-6-methylindole* (**4a)**: Yellow solid, yield 73%, m.p. 132–134 °C. ^1^H-NMR (400 MHz, CDCl_3_) *δ*: 8.23 (d, *J* = 8.4 Hz, 1H), 8.18 (s, 1H), 7.95–7.97 (m, 2H), 7.91 (s, 1H), 7.58–7.60 (m, 1H), 7.47–7.51 (m, 2H), 7.31 (d, *J* = 8.4 Hz, 1H), 3.59 (s, 2H), 2.57 (s, 3H), 2.34 (s, 4H), 1.55–1.60 (m, 4H), 1.43–1.44 (m, 2H). ^13^C-NMR (125 MHz, CDCl_3_) *δ*: 193.4, 137.5, 137.0, 135.1, 134.5, 132.0, 129.5, 127.1, 126.4, 126.3, 122.5, 121.8, 113.3, 63.7, 54.3, 27.8, 26.0, 24.3. MS (ESI-TRAP), *m/z* (%): 397 ([M+H]^+^, 100). HRMS (ESI): Calcd for C_22_H_25_N_2_O_3_S ([M+H]^+^), 397.1580; found, 397.1571.

*N-Phenylsulfonyl-3-[2-morpholinoethanone]-6-methylindole* (**4b**): White solid, yield 66%, m.p. 174–176 °C. ^1^H-NMR (500 MHz, CDCl_3_) *δ*: 8.25 (d, *J* = 8.0 Hz, 1H), 8.19 (s, 1H), 7.94–7.96 (m, 2H), 7.90 (s, 1H), 7.62 (t, *J* = 7.5 Hz, 1H), 7.48–7.51 (m, 2H), 7.33 (dd, *J* = 8.0 Hz, 1.0 Hz, 1H), 3.71 (t, *J* = 4.5 Hz, 4H), 3.61 (s, 2H), 2.57 (s, 3H), 2.41 (t, *J* = 4.0 Hz, 4H). ^13^C-NMR (125 MHz, CDCl_3_) *δ*: 193.4, 137.5, 136.2, 135.1, 134.5, 132.1, 129.5, 127.1, 126.7, 126.2, 122.8, 121.7, 113.3, 67.0, 63.3, 53.4, 27.8. MS (ESI-TRAP), *m/z* (%): 399 ([M+H]^+^, 100). HRMS (ESI): Calcd for C_21_H_23_N_2_O_4_S ([M+H]^+^), 399.1373; found, 399.1361.

*N-Phenylsulfonyl-3-[2-pyrrolidinoethanone]-6-methylindole* (**4c**): Yellow solid, yield 57%, m.p. 124–126 °C. ^1^H-NMR (500 MHz, CDCl_3_) *δ*: 8.24 (d, *J* = 8.5 Hz, 1H), 8.18 (s, 1H), 7.95–7.97 (m, 2H), 7.91 (s, 1H), 7.58–7.61 (m, 1H), 7.47–7.50 (m, 2H), 7.33–7.35 (m, 1H), 3.75 (s, 2H), 2.56 (s, 3H), 2.49 (s, 4H), 1.78–1.80 (m, 4H). ^13^C-NMR (125 MHz, CDCl_3_) *δ*: 193.3, 137.5, 135.1, 134.5, 132.0, 129.5, 127.1, 126.5, 126.1, 122.7, 121.8, 113.2, 60.5, 53.8, 27.7, 23.5. MS (ESI-TRAP), *m/z* (%): 383 ([M+H]^+^, 100). HRMS (ESI): Calcd for C_21_H_23_N_2_O_3_S ([M+H]^+^), 383.1424; found, 383.1417.

*N-Phenylsulfonyl-3-[2-diisopropylaminoethanone]-6-methylindole* (**4d**): Tan liquid, yield 49%. ^1^H-NMR (500 MHz, CDCl_3_) *δ*: 8.17–8.19 (m, 2H), 8.02 (s, 1H), 7.97 (d, *J* = 8.0 Hz, 2H), 7.57–7.60 (m, 1H), 7.46–7.49 (m, 2H), 7.32 (d, *J* = 8.0 Hz, 1H), 3.74 (s, 2H), 2.99–3.04 (m, 2H), 2.56 (s, 3H), 1.03 (d, *J* = 6.5 Hz, 12H). ^13^C-NMR (125 MHz, CDCl_3_) *δ*: 193.4, 142.0, 137.6, 135.3, 134.4, 131.6, 129.5, 127.2, 126.0, 124.9, 122.2, 121.8, 112.1, 49.0, 48.0, 27.7, 20.8. MS (ESI-TRAP), *m/z* (%): 413 ([M+H]^+^, 100). HRMS (ESI): Calcd for C_23_H_29_N_2_O_3_S ([M+H]^+^), 413.1893; found, 413.1890.

*N-Phenylsulfonyl-3-[2-butylaminoethanone]-6-methylindole* (**4e**): Yellow liquid, yield 51%. ^1^H-NMR (500 MHz, CDCl_3_) *δ*: 8.26 (d, *J* = 8.5 Hz, 1H), 8.18 (s, 1H), 7.95–7.97 (m, 2H), 7.90 (s, 1H), 7.59–7.62 (m, 1H), 7.48–7.51 (m, 2H), 7.31–7.33 (m, 1H), 3.91 (s, 2H), 2.75 (s, 1H), 2.58–2.61 (m, 2H), 2.56 (s, 3H), 1.48–1.51 (m, 2H), 1.32–1.36 (m, 2H), 0.89–0.92 (m, 3H). ^13^C-NMR (125 MHz, CDCl_3_) *δ*: 193.4, 138.7, 137.6, 135.2, 134.5, 132.0, 129.6, 127.0, 126.4, 125.4, 122.9, 121.8, 112.4, 53.9, 48.8, 32.1, 27.7, 20.4, 14.0. MS (ESI-TRAP), *m/z* (%): 385 ([M+H]^+^, 100). HRMS (ESI): Calcd for C_21_H_25_N_2_O_3_S ([M+H]^+^), 385.1580; found, 385.1572.

*N-(p-Ethyl)phenylsulfonyl-3-[2-morpholinoethanone]-6-methylindole* (**4f**): White solid, yield 37%, m.p. 108–110 °C. ^1^H-NMR (500 MHz, CDCl_3_) *δ*: 8.25 (d, *J* = 8.5 Hz, 1H), 8.19 (s, 1H), 7.92 (s, 1H), 7.87 (d, *J* = 8.0 Hz, 2H), 7.29–7.33 (m, 3H), 3.71 (s, 4H), 3.62 (s, 2H), 2.68(q, *J* = 7.5 Hz, 2H), 2.56 (s, 3H), 2.42 (s, 4H), 1.21 (t, *J* = 7.5 Hz, 3H). ^13^C-NMR (125 MHz, CDCl_3_) *δ*: 193.4, 151.9, 135.1, 134.7, 132.2, 129.0, 127.3, 126.7, 126.2, 122.8, 121.5, 113.4, 67.0, 63.3, 53.4, 28.8, 27.7, 14.8. MS (ESI-TRAP), *m/z* (%): 427 ([M+H]^+^, 100). HRMS (ESI): Calcd for C_23_H_27_N_2_O_4_S ([M+H]^+^), 427.1686; found, 427.1677.

### 3.3. General Procedure for the Synthesis of **6**

Firstly, intramolecular cyclization of benzoylhydrazine with carbon disulfide and potassium hydroxide in the presence of ethanol resulted in 5-phenyl-1,3,4-oxadiazole-2-thiol (**5**) [10]. Subsequently, a mixture of **5** (0.5 mmol), **3b** (0.6 mmol) and K_2_CO_3_ (1 mmol) in acetone (5 mL) was reacted at reflux, and the reaction process was checked by TLC. When the reaction was complete after 9 h, the organic solvent was removed, and the residue was directly purified by preparative TLC to give **6**. The structure of the compound was well characterized by ^1^H-NMR, ^13^C-NMR, m.p., and MS.

*N-(p-Ethyl)phenylsulfonyl-3-[2-(5-phenyl-1,3,4-oxadiazole-2-yl-thio)ethanone]-6-methylindole* (**6**): White solid, yield 36%, m.p. 122–124°C. ^1^H-NMR (500 MHz, CDCl_3_) *δ*: 8.29 (d, *J* = 8.0 Hz, 1H), 8.18 (s, 1H), 8.15 (s, 1H), 7.98–8.00 (m, 2H), 7.90 (d, *J* = 8.5 Hz, 2H), 7.49–7.52 (m, 3H), 7.43 (d, *J* = 7.5 Hz, 1H), 7.29–7.41 (m, 2H), 4.64 (s, 2H), 2.58–2.59 (m, 2H), 2.54 (s, 3H), 1.13 (t, *J* = 7.5 Hz, 3H). ^13^C-NMR (125 MHz, CDCl_3_) *δ*: 193.2, 165.8, 163.6, 151.9, 134.9, 134.4, 133.7, 132.5, 131.7, 129.2, 129.1, 129.0, 127.3, 127.2, 126.6, 125.9, 123.6, 123.4, 121.4, 114.0, 37.0, 28.8, 27.7, 14.6. MS (ESI-TRAP), *m/z* (%): 518 ([M+H]^+^, 100). HRMS (ESI): Calcd for C_27_H_24_N_3_O_4_S_2_ ([M+H]^+^), 518.1203; found, 518.1211.

### 3.4. Anti-HIV-1 Activity Assay

#### 3.4.1. Cells and Virus

Cell line (C8166) and the laboratory-derived virus (HIV-1 _IIIB_) were obtained from the MRC, AIDS Reagent Project, London, UK. C8166 was maintained in RPMI-1640 supplemented with 10% heat-inactivated new born calf serum (Gibco, USA). The cells used in all experiments were in log-phase growth. The 50% HIV-1 _IIIB_ tissue culture infectious dose (TCID_50_) in C8166 cells was determined and calculated by the Reed and Muench method. Virus stocks were stored in small aliquots at −70 °C [11].

#### 3.4.2. MTT-based Cytotoxicity Assay

Cellular toxicity of compounds **4a**–**f** and **6** on C8166 cells was assessed by the MTT method as described previously [12]. Briefly, cells were seeded on 96-well microtiter plate in the absence or presence of various concentrations of compounds in triplicate and incubated at 37 °C in a humid atmosphere of 5% CO_2_ for 3 days. The supernatants were discarded and MTT reagent (5 mg/mL in PBS) was added to each wells, then incubated for 4 h, 100 μL of 50% DMF-20% SDS was added. After the formazan was dissolved completely, the plates were read on a Bio-TekElx800 ELISA reader at 595/630 nm. The cytotoxic concentration that caused the reduction of viable C8166 cells by 50% (CC_50_) was determined from dose-response curve.

#### 3.4.3. Syncytia Assay

In the presence of 100 μL of various concentrations of compounds, C8166 cells (4 × 10^5^/mL) were infected with virus HIV-1_IIIB_ at a multiplicity of infection (M.O.I) of 0.06. The final volume per well was 200 μL. Control assays were performed without the testing compounds in HIV-1_IIIB_ infected and uninfected cultures. After 3 days of culture, the cytopathic effect (CPE) was measured by counting the number of syncytia. Percentage inhibition of syncytia formation was calculated and 50% effective concentration (EC_50_) was calculated. AZT (Sigma, USA) was used as a positive control. Therapeutic index (TI) = CC_50_/EC_50_ [13].

## 4. Conclusions

In summary, a series of *N*-arylsulfonyl-3-(2-yl-ethanone)-6-methylindolederivatives **4a**–**f** and **6** were easily synthesized and have been identified as inhibitors of HIV-1 replication. Initial biological studies indicated that *N*-(p-ethyl)phenylsulfonyl-3-[2-morpholinoethanone]-6-methylindole (**4f**) and *N*-(*p*-ethyl)phenylsulfonyl-3-[2-(5-phenyl-1,3,4-oxadiazole-2-ylthio)-ethanone]-6-methylindole (**6**) show promising activity against HIV-1 replication. Our structure-based design has led to the discovery that the introduction of an ethyl group at the *para*-position of the arylsulfonyl ring generated a novel class of highly potent anti-HIV-1 compounds. Moreover, the preliminary SAR showed that the morpholino group or the 5-phenyl-1,3,4-oxadiazole-2-thiol group at the C-2 position on the acetyl of *N*-arylsulfonyl-3-(2-yl-ethanone)-6-methylindoles was very important for potent anti-HIV-1 activity. The EC_50_ and TI values of **4f** and **6** were 9.42/4.62 μM, and >49.77/66.95, respectively. The application of this novel and potent structure modification is expected to provide the foundation for the rational modification of *N*-arylsulfonyl-3-acetylindoles and accelerate the discovery of more potent anti-HIV-1 compounds.
